# Seasonal dynamics of bacterial community structure and function in the surf zone seawater of a recreational beach in Ostend, Belgium

**DOI:** 10.1111/1758-2229.70031

**Published:** 2024-11-08

**Authors:** Yunmeng Li, Pascal I. Hablützel, Zixia Liu, Emmanuel Van Acker, Colin R. Janssen, Jana Asselman, Maarten De Rijcke

**Affiliations:** ^1^ Flanders Marine Institute (VLIZ) InnovOcean Campus Ostend Belgium; ^2^ Blue Growth Research Lab Ghent University Ostend Belgium; ^3^ Department of Biology Vrije Universiteit Brussel Brussels Belgium; ^4^ Laboratory of Environmental Toxicology and Aquatic Ecology (GhEnToxLab), Department of Animal Sciences and Aquatic Ecology Ghent University Ghent Belgium; ^5^ Present address: Witteveen+Bos Belgium N.V. Antwerpen‐Berchem Belgium

## Abstract

Despite the importance of bacteria in surf zone water quality, detailed insights into their community composition, functions, and seasonal dynamics at recreational beaches are scarce. This study conducted year‐long, weekly monitoring of bacterial communities and environmental factors at a recreational beach in Ostend, Belgium. Using full‐length 16S rRNA gene sequencing, we correlated bacterial composition and predicted functions with environmental factors to identify potential drivers. Bacterial communities were significantly affected by seasonal variations in chlorophyll *a* (Chl *a*), net primary productivity (NPP), and seawater temperature (SWT), with minimal influence from faecal inputs due to human activities. Spring showed distinct abundances of *Planktomarina*, *Amylibacter*, and *Sulfitobacter*, positively correlated with Chl *a* and related to sulphur oxidation potential. Summer had higher abundances of Cryomorphaceae, likely enhancing chemoheterotrophy. Beginning in mid to late fall and extending into winter, bacterial communities underwent substantial changes. Fall featured a distinctive enrichment of Thioglobaceae, inversely correlated with Chl *a*. Winter was dominated by Methylophilaceae (OM43 clade), negatively correlated with Chl *a*, NPP, and SWT. Both seasons exhibited elevated levels of potentially pathogenic phenotypes and predicted functions related to methanol oxidation and methylotrophy. This study provides a baseline for understanding how surf zone bacterial communities respond to environmental changes and impact health.

## Graphical abstract

This study involved year‐long, weekly monitoring of bacterial community composition and predicted functions in the surf zone seawater of a recreational beach in Ostend, Belgium, using full‐length 16S rRNA gene sequencing. The data were then correlated with environmental factors to identify potential drivers. Bacterial communities were significantly affected by seasonal variations in chlorophyll *a* (Chl *a*), net primary productivity (NPP), and seawater temperature (SWT), with minimal influence from faecal inputs due to beach recreational activities, as indicated by the ratio of *Bacilli*, *Bacteroidota*, and *Clostridia* to *Alphaproteobacteria* (BBC:A). This study provides a baseline for understanding how surf zone bacterial communities respond to environmental changes and impact health.

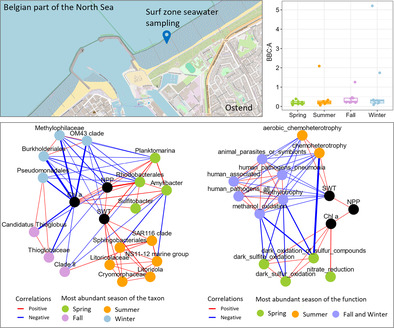

## INTRODUCTION

Coastal areas, popular for their vibrant beach culture and affordable opportunities for water sports, attract many visitors for recreational activities such as swimming and surfing. The quality of coastal seawater, particularly in surf zones, is directly linked to public health and the well‐being of coastal ecosystems (WHO, 2021). Bacteria in seawater play pivotal roles in elemental cycling and energy flow, essential for maintaining ecosystem functions and services (Charlson et al., 2007; Mou et al., 2008; Voss et al., 2013). Moreover, these bacteria can impact human health through various exposure routes (e.g., skin contact, ingestion, and inhalation), offering benefits like enhancing immunity or posing risks by causing infections and diseases (Landrigan et al., 2020; Li et al., 2024; Pendergraft et al., 2022; Santos et al., 2020). Consequently, understanding the community structure and function of bacteria in surf zone seawater is crucial for ensuring the sustainability of coastal ecosystems and safeguarding public health.

Research on bacteria in surf zone seawater has primarily focused on routine monitoring of common pathogens and faecal indicators, such as *Enterococcus* and *Escherichia coli* (Rosenfeld et al., 2006; Şanlıtürk & Güran, 2022). Studies have also examined the changes in these organisms and broader bacterial communities in response to natural and human‐induced factors that could affect water quality. These factors include land‐sea runoff from stormwater, discharge of polluted river water, and untreated or leaking sewage (Li et al., 2022; Orel et al., 2022; Pendergraft et al., 2022), as well as beach nourishment (also known as sand replenishment or beach fill) (Rippy et al., 2013) and the widespread presence of beach plastic litter (De Tender et al., 2015). Typically, these investigations compared bacterial profiles (number and composition) at their studying sites against World Health Organization water quality standards or benchmarks from potential pollution sources, using offshore seawater as a negative control. These studies provide valuable insights into how bacterial communities respond to external perturbations and help assess health risks. However, the composition and function of bacterial communities under ‘normal’ conditions—free from distinct pollution factors and disease outbreaks—in surf zone seawater remain less studied. This gap is particularly relevant given the widely reported better health of living at or frequently visiting the coast by epidemiological studies (Geiger et al., 2023; Hooyberg et al., 2020; Wheeler et al., 2012), and the increasing attention to the potential contributions from marine bioactive molecules exposure via the inhalation of sea spray aerosols (Asselman et al., 2019; Li et al., 2024; Moore, 2015; Van Acker et al., 2020; Van Acker et al., 2021).

Temporal (from hours to seasons) and spatial (both vertical and horizontal) variations in the structure of bacterial communities in coastal seawater have been extensively documented. Bacterial community composition is influenced by distinct environmental gradients such as light, water temperature, nutrients, and water mass (Bunse & Pinhassi, 2017; Fortunato et al., 2012; Zhao et al., 2019). The surf zone is often characterized by intense light and harsh hydrodynamic conditions due to breaking waves interacting with coastal topography, leading to continuous sediment mixing and high turbidity. Studies on phytoplankton and zooplankton dynamics within the surf zone have shown that these communities are influenced by factors such as wave height, water temperature, suspended particulate matter, and chlorophyll *a* (Chl *a*), and can contain species that are unique to this environment (Guerreiro et al., 2021; Menéndez et al., 2019; Shanks et al., 2018). However, the dynamics of bacterial communities in surf zone seawater and their environmental drivers are underexplored.

This study aims to investigate the structure and function of bacterial communities in surf zone seawater and to identify their environmental drivers. The chosen study site is the surf zone of a recreational beach in Ostend, Belgium, which features a temperate climate with warm summers, cold winters, and moderate springs and falls. This beach is a popular destination for various recreational activities, particularly during the summer. No severe pollution event or disease outbreak has been connected to this site in the past. Considering that the local water residence time is around 7 days (Belgische Staat, 2018), we collected surface seawater from the surf zone at weekly intervals over the course of a year. Bacterial composition was analyzed using Nanopore full‐length 16S rRNA gene sequencing, which provides a higher number of sequence assignments and taxa identification at the genus and species levels compared to short‐read sequencing (Matsuo et al., 2021; Szoboszlay et al., 2023). Bacterial functions were predicted by the Functional Annotation of Prokaryotic Taxa (FAPROTAX) (Louca et al., 2016). Both abiotic factors (e.g., seawater temperature, wave height, and solar radiation) and biotic factors (e.g., Chl *a* and net primary productivity) capturing seasonal variations were monitored and then correlated with the bacterial community structures and predicted functions to identify key environmental drivers. The results allow us to better understand the ecological roles of bacterial communities in the surf zone and their potential impacts on environmental health and public safety.

## EXPERIMENTAL PROCEDURES

### Sample collection and environmental monitoring

Between March 2018 and March 2019, weekly surface seawater samples were collected at knee‐deep depth in the surf zone of a recreational sandy beach in Ostend, Belgium (about 51°14′28″N, 2°55′56″ E), using 1 L glass bottles. We used the astronomical equinoxes and solstices to define the seasons. Specifically, spring was defined as 20 March 2018–20 June 2018; summer as 21 June 2018–22 September 2018; fall as 23 September 2018–20 December 2018; and winter as 21 December 2018–19 March 2019. Each season, a total of 13 samples were collected, resulting in 52 samples over the year. After collection, samples were immediately transported to the laboratory at the Marine Station Ostend (51°14′10″N, 2°55′42″ E; about 700 m from the sampling site) for processing. The samples were first filtered through a 60 μm nylon mesh to remove large debris, then passed through a 10 μm PTFE filter (Ø 47 mm, Omnipore) to eliminate finer particles, and finally through a 0.1 μm PTFE filter (Ø 47 mm, Omnipore) to capture bacteria. The 0.1 μm filters were immediately sealed in petri dishes (Ø 47 mm, Merck) and stored at −86°C until DNA extraction. All contacting materials were either single‐use or sterilized with 70% ethanol (Chem‐Lab) and thoroughly rinsed with Milli‐Q water (Merck‐Millipore) before use.

Seawater parameters, including seawater temperature, wave height, and wave period, were measured every 30 min by the ‘Ostend eastern palisade–Buoy’ (51°14′48″N, 2°55′39″ E; about 600 m from the sampling site) and sourced from the public database ‘*Meetnet Vlaamse Banken’* (https://meetnetvlaamsebanken.be/). Surface seawater productivity data, including Chl *a* and net primary production (NPP) estimates, were obtained from Oregon State University's Ocean Productivity database (http://sites.science.oregonstate.edu/ocean.productivity/index.php) using R package nppr (Xu, 2023). The Chl *a* and NPP estimates were 8‐day averaged data with a spatial resolution of 0.1° from Moderate‐resolution Imaging Spectroradiometer aqua satellite. The NPP data was calculated using the Vertically Generalized Productivity Model. Meteorological conditions including air temperature, solar radiation, relative humidity (RH), and precipitation from remote observations (satellite and radar) were downloaded from Visual Crossing Weather Data (https://www.visualcrossing.com/). Detailed sample information and environmental variables are provided in Table S1 and Figure S1.

### DNA extraction and nanopore full‐length 16S rRNA gene sequencing

Total DNA was extracted from whole filters using the Qiagen DNeasy Blood & Tissue Kit, with some modifications to the manufacture's protocol. Specifically, filters stored in petri dishes were first transferred to 2 mL tubes (Precellys Lysing Kit) using sterilized tweezers. An initial bead‐beating step was then performed by adding 0.5 g of 0.1 mm glass beads (BioSpec Products) and 500 μL ATL Buffer (Qiagen) to each tube, followed by shaking in a Minilys homogenizer (Bertin Technologies) at maximum speed for 45 s. After this, the samples were incubated at 56°C for 30 min on a revolver rotator (model No. 88881002, Thermo Fisher Scientific) inside an incubator (My Temp mini digital incubators, Benchmark Scientific). This entire process, including the shaking and incubation steps, was repeated with an additional 500 μL of ATL Buffer. Next, 55.56 μL of Proteinase K (final concentration 2 mg/L; Qiagen) was added to each tube. The tubes were then shaken for 10 seconds and incubated at 56°C for 2 hours. Following this, the samples were shaken for 15 seconds and centrifuged at 4000 rcf for 1 min. The supernatant from each tube was transferred to a new 1.5 mL centrifuge tube (Eppendorf) and centrifuged at 13,000 rcf for 1 min. Subsequently, 325 μL of the bead‐free supernatant was transferred into a new 2 mL tube. The manufacturer's protocol was followed thereafter with the following modifications: 325 μL of AL buffer was used instead of 200 μL during the lysis step, 325 μL of 96% ethanol instead of 200 μL during the precipitation step, 250 μL each of AW1 and AW2 buffers instead of 500 μL during the washing steps, and a final elution step of 25 μL AE buffer instead of 200 μL for each sample. One lab‐negative control was included during each batch of DNA extractions. Extracted DNA was quantified using the Qubit dsDNA HS (high sensitivity) assay kit with a Qubit 3.0 fluorometer (Thermo Fisher Scientific).

Library preparation was conducted using the SQK‐16S024 16S barcoding kit 1‐24 (Oxford Nanopore Technologies) and the ‘rapid sequencing amplicons—16S barcoding protocol’ version 16S_9086_v1_revU_14Aug2019 with some modifications. Briefly, a 25 μL PCR reaction system included 12.5 μL MyTaq 2× master mix (Bioline), 5 μL of one barcoded 16S primer pair, 5 μL DNA and 2.5 μL nuclease‐free water. The barcoded 16S primer pairs used were 27F and 1492R to amplify the full‐length 16S rRNA gene. The PCR was conducted as follows: denaturation at 94°C for 180 s, 35 cycles of 94°C for 30 s, 50°C for 60 s, 72°C for 90 s, and a final extension at 72°C for 30 s. The PCR product was run on 1% agarose gel stained by SYBR Green I Nucleic Acid Gel Stain (Invitrogen) to check for amplification success and unspecific reactions. The PCR product was then cleaned using 1.8× CleanPCR reagent (GC Biotech) with a 5 min incubation on a revolver rotator (model No. 88881002, Thermo Fisher Scientific) and three washes with 70% ethanol on a magnetic stand (DynaMag‐2, Invitrogen), followed by a final elution in 10 μL of 10 mM Tris–HCl (pH 8.0, with 50 mM NaCl). The final concentration of PCR products was measured and then pooled at 6 ng per sample. When a sample's DNA concentration was too low, the maximum amount of DNA was added. Pools (7 in total) were again quantified and a total of 50–100 ng DNA was taken from the pools into a new tube and diluted in 10 mM Tris–HCl (pH 8.0, with 50 mM NaCl) to obtain a total volume of 10 μL per library. The 10 μL library then had 1 μL of rapid adapter (RAP) added to it for a 5 min incubation step, then was diluted with 34 μL sequencing buffer, 25.5 μL loading beads, and 4.5 μL nuclease‐free water to get 75 μL library finally. The 75 μL library was loaded on the FLO‐MIN106D R9 flow cell for sequencing.

Nanopore sequencing using a MinION Mk1B device was conducted on a laptop computer which permits base‐calling with Guppy (version 5.1.13). Sequencing was initiated through MinKNOW (version 21.11.7) with the fast base‐calling model and a *Q* score of ≥7, enabling quick monitoring of sequence output in approximately real‐time. Once sequencing was complete, the raw FAST5 files were re‐base‐called using the high‐accuracy base‐calling model with barcode removal to obtain high‐quality, demultiplexed FASTQ data for subsequent processing and analysis.

### Sequencing data processing and statistical analysis

FASTQ data were filtered with a *Q* score of ≥8 and a read length of 1400–1700 bp using NanoFilt (version 2.8.0) (De Coster et al., 2018). After filtering, sequences were error‐corrected and clustered using the density‐based clustering approach ordering points to identify the clustering structure, and consensus sequences for each cluster were generated through multi‐sequence alignment using SPOA, both utilizing ASHURE (Baloğlu et al., 2021) with 10 iterations. Chimeric sequences in both the filtered raw sequences and the generated consensus sequences were detected and removed using VSEARCH (version 2.22.1) (Rognes et al., 2016). The resulting non‐chimeric consensus sequences (which are equivalent to amplicon sequence variants) were then taxonomically assigned using BLASTn (version 2.13.0) (Camacho et al., 2009) against the SILVA 138.1 database, with a threshold of 90% identity and an *e*‐value of 1e^−10^. The BLASTn hits with the highest percentage of identity and an alignment length ≥1200 bp were selected, including details such as query ID (i.e., the non‐chimeric consensus sequence), subject ID, percentage of identity, alignment length, *e*‐value, bit score, and taxonomy. The filtered non‐chimeric raw sequences were mapped onto the non‐chimeric consensus sequences through global alignment using Minimap2 (version 2.24) (Li, 2018) to obtain counts for each consensus sequence, with each sequence assigned to the consensus sequence with which it had the highest matching percentage. Since all these processes were conducted on a sample‐by‐sample basis, the query IDs of non‐chimeric consensus sequences were not universal across samples. However, different consensus sequences from the same or different samples might align to the same reference sequence in the SILVA v138.1 database, resulting in the same subject ID. Thus, to ensure consistent and comparable classification of operational taxonomic units (OTUs) across samples, we used the subject ID obtained from BLASTn results as the OTU identifier. This approach ensures that sequences from different samples that align to the same reference sequence are classified under the same OTU, providing a reliable basis for downstream comparative analysis. OTUs classified as chloroplast, mitochondria, or those lacking phylum‐level annotation were discarded. OTUs with fewer than 5 reads were also removed to reduce noise. The final sequence counts for each sample, used for subsequent analysis, are provided in Table S1.

Rarefaction curves, based on observed OTUs, were generated to assess the sufficiency of sequencing depth in capturing bacterial diversity. The sequences were then randomly rarefied to the least number in all samples per sample for alpha diversity analysis. Alpha diversity indices, including richness (i.e., observed OTUs), evenness (i.e., Pielou index), and Shannon index, were calculated to evaluate the diversity within each sample using the vegan package in R (version 4.2.3). A Bray–Curtis distance matrix of total OTU relative abundance was calculated to evaluate bacterial community dissimilarity among samples using the vegan package and visualized using principal coordinate analysis (PCoA) with the ape package. Prior to these analyses, overdispersion in the dataset was assessed by examining the variance‐to‐mean ratio of the total OTU relative abundances across samples, ensuring that the data variability was adequately accounted and did not introduce bias into the community analysis. Permutational multivariate analysis of variance (PERMANOVA; ‘*adonis2*’ function) using the Bray–Curtis dissimilarity matrix with 999 permutations was performed to test community differences among seasons. According to the taxonomic information, bacterial community composition was calculated at different taxonomic levels. Linear discriminant analysis (LDA) effect size (LEfSe) was used to determine the taxa with significant differences across seasons (Segata et al., 2011), with the LDA score set to 4 and the taxa set from phylum to genus.

Bacterial phenotypic traits, including Gram‐positive, Gram‐negative, potentially pathogenic, aerobic, anaerobic, facultatively anaerobic, stress‐tolerant, biofilm‐forming, and mobile element‐containing, were predicted using BugBase (https://bugbase.cs.umn.edu/index.html) (Ward et al., 2017). BugBase requires a 16S OTU table aligned against the Greengenes database, as it uses these OTU IDs to predict phenotypes based on the functional and phenotypic annotations linked to them. To perform phenotypic inference, the FASTQ data were re‐processed using the same method described earlier, with the only difference being that the sequences were aligned against the Greengenes v13_5 database instead of the SILVA v138.1 database. For the thresholds that determine whether an OTU exhibits a specific phenotype, we used the BugBase's default setting. The setting automatically adjusts the thresholds based on the observed variance (i.e., the degree of variation in a particular phenotypic trait across all samples), selecting the threshold with the highest variance. This approach helps to identify phenotypic traits that vary the most between samples, making it easier to detect meaningful differences in the bacterial communities. Bacterial ecological and metabolic functions were predicted using the FAPROTAX, which is based on the current prokaryotic function annotation library of cultivable bacteria and is well‐suited for functional annotation and prediction of environmental samples (Louca et al., 2016).

Potential impacts of beach recreational activities, particularly faecal inputs, on bacterial communities, were evaluated using the relative abundance ratio of *Bacilli*, *Bacteroidota*, and *Clostridia* (often abundant in faecal samples) to *Alphaproteobacteria* (less abundant in faecal samples) (BBC:A) (Wu et al., 2010). The effects of environmental variables (Euclidean distance matrix) on bacterial composition and predicted functions (Bray–Curtis distance matrices) were tested using the Mantel test with Pearson's correlation (the linkET package). Prior to this analysis, the environmental variables were standardized using the ‘*scale*’ function. Following this, the correlations between seasonally significant taxa, identified by LEfSe, and the 15 most abundant functions, with environmental variables found significant in the Mantel test, were visualized through co‐occurrence networks with the igraph package.

The heterogeneity of environmental conditions across seasons was evaluated using a principal component analysis (PCA; ‘*prcomp*’ function) based on the covariance matrix of standardized environmental variables. Differences in environmental variables, alpha diversity, bacterial composition, predicted phenotypes, and functions across seasons were tested using the Kruskal–Wallis rank test, followed by the Wilcox test for post‐hoc comparisons. The significance level of 0.05 was used.

## RESULTS

### Successfully sequenced samples and environmental conditions across seasons

The rarefaction curves for 35 out of the 52 collected samples reached a plateau, suggesting that the sequencing depth for these samples sufficiently captured most of the OTUs necessary for diversity analysis (Figure S1A, B, Table S1). The 35 samples included 9 from spring, 8 from summer, 9 from fall, and 9 from winter. Due to irregular sampling intervals among these 35 samples across different seasons (Figure S1A), we conducted a PCA based on the covariance matrix of environmental factors to assess whether these samples could adequately represent the overall environmental variability observed in the complete set of 52 samples. The values of Chl *a* and net primary productivity (NPP) were unavailable for 10 samples (5 each in fall and winter) (Figure S1A); therefore, only 42 samples (13 each in spring and summer, 8 each in fall and winter) represent the complete set, and 26 samples (9 in spring, 8 in summer, 4 in fall, and 5 in winter) from the successfully sequenced set, were included in the PCA. The PCA results indicated that despite the irregular intervals, the 26 samples effectively captured the environmental variability (Figure S1D), closely mirroring the patterns observed in the 42 samples (Figure S1C).

Table 1 displays the average environmental conditions during sampling among different seasons of the year, calculated based on the 26 samples. Spring and summer showed higher levels of Chl *a* and NPP compared to fall and winter (Wilcox, all *p* <0.05). Chl *a* levels were relatively high in spring (10.7 ± 4.2 mg/m^3^) compared to summer (6.5 ± 1.7 mg/m^3^) (Wilcox, *p* = 0.11). Summer exhibited the highest seawater temperature (SWT) and air temperature (AT), followed by spring and fall (Wilcox, both *p* <0.05), with winter having the lowest SWT and AT (Wilcox, all *p* <0.05). Fall exhibited lower solar radiation (SR) levels compared to spring and summer (Wilcox, both *p* <0.05). SR in winter did not significantly differ from the other three seasons (Wilcox, all *p* >0.05). No significant differences in wave height, wave period, RH, and precipitation were observed among seasons (Kruskal–Wallis, all *p* >0.05). The inclusion of another 5 fall and 4 winter samples (35 in total), which were successfully sequenced but lacked Chl *a* and NPP data, did not distinctly affect the changes of other environmental variables across seasons (Table S2). Consequently, all 35 samples that passed sequencing were subsequently used to assess bacterial community structure and function across seasons. Of these, only the 26 samples with complete environmental data were used to investigate the effects of environmental variables on bacterial composition and functions.

**TABLE 1:** Average environmental conditions during sampling among different seasons of the year (Mean ± Standard deviation (SD)).

Variables	Spring (*n* = 9)	Summer (*n* = 8)	Fall (*n* = 4)	Winter (*n* = 5)
SWT (°C)	13.7 ± 4.6^a^	20.1 ± 1.1^b^	13.5 ± 2.1^ac^	6.7 ± 1.6^d^
WH (cm)	61.6 ± 27.4	77.6 ± 36.4	82.8 ± 22.3	61.2 ± 14.9
WP (s)	3.6 ± 0.6	3.6 ± 0.5	4.8 ± 0.9	3.9 ± 0.7
Chl *a* (mg/m^3^)	10.7 ± 4.2^a^	6.5 ± 1.7^a^	4.5 ± 0.4^b^	4.0 ± 1.0^b^
NPP (mg C/m^2^/day)	6841.3 ± 2889.0^a^	7033.2 ± 1765.1^ac^	2578.8 ± 814.4^b^	1387.7 ± 537.3^b^
AT (°C)	13.9 ± 3.9^a^	18.8 ± 2.0^b^	12.3 ± 2.5^a^	6.3 ± 2.8^c^
SR (W/m^2^)	363.8 ± 163.8^a^	379.7 ± 239.5^a^	63.0 ± 50.3^b^	236.5 ± 180.6^ab^
RH (%)	83.3 ± 6.7	78.1 ± 8.2	83.8 ± 2.2	78.3 ± 8.4
Precip. (mm)	0.0 ± 0.0	0.1 ± 0.2	0.8 ± 1.3	0.0 ± 0.0

*Note*: The superscripts present the results of Wilcox test, and the data with different superscripts in the same row are significantly different (*p* < 0.05).

Abbreviations: AT, air temperature; Chl *a*, chlorophyll *a*; NPP, net primary productivity; Precip., precipitation; RH, relative humidity; SR, solar radiation; SWT, seawater temperature; WH, wave height; WP, wave period.

### Seasonal dynamics of bacterial diversity and composition

**FIGURE 1:**
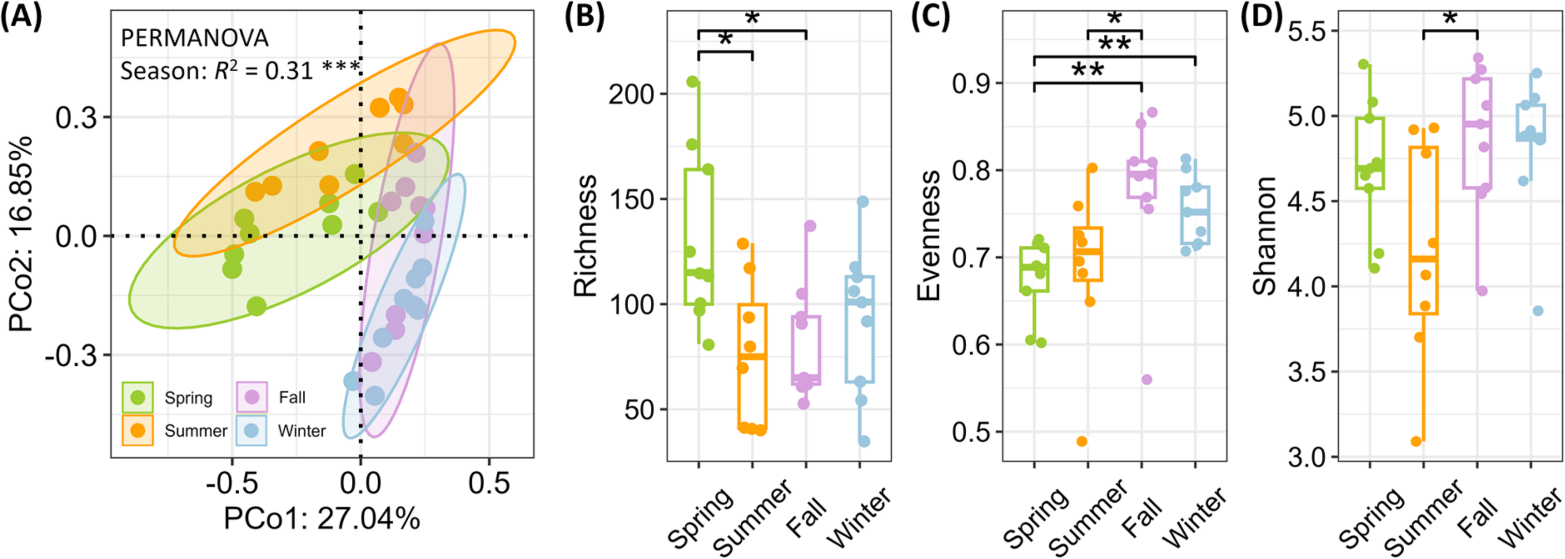
(A) A principal coordinate analysis (PCoA) and PERMANOVA test of bacterial communities among different seasons based on Bray–Curits distances using OTU relative abundance. ***, *p* <0.001. Boxplot of alpha diversity indices, including **(**B) richness, (C) evenness, and (D) Shannon index, across seasons. Differences among seasons were tested using the Kruskal–Wallis test, followed by the Wilcox test for post‐hoc comparisons. *, *p* <0.05; **, *p* <0.01.

Differences in bacterial community structure among samples, as indicated by Bray–Curtis distances based on OTU relative abundance, were visualized using a PCoA (Figure 1A). The PCoA, along with a PERMANOVA test, showed that seasonal changes significantly influenced the structure of bacterial communities (‘*adonis2*’, *R* ^2^ = 0.31, *p* <0.001). Spring and summer samples were more closely related, as were fall and winter samples.

The richness and diversity of bacteria within each sample were quantified using alpha diversity indices, and the results were visualized across different seasons (Figure 1B–D). Richness was highest in spring compared to other seasons (Figure 1B, Wilcox, *p* <0.05 for summer and fall; *p* = 0.1 for winter). In contrast, evenness was lowest in spring compared to other seasons (Figure 1C, Wilcox, *p* = 0.32 for summer; *p* <0.01 for fall and winter). The Shannon index, which integrates both richness and evenness, did not exhibit significant differences between spring and other seasons (Figure 1D, Wilcox, all *p* >0.05). Summer and fall exhibited similar richness levels (Figure 1B, Wilcox, *p* = 0.77), while fall displayed higher evenness than summer (Figure 1C, Wilcox, *p* <0.05), resulting in a higher Shannon index in fall (Figure 1D, Wilcox, *p* <0.05). No significant differences in alpha diversity indices between summer and winter, or between fall and winter were observed (Figure 1B–D, Wilcox, all *p* >0.05).

**FIGURE 2:**
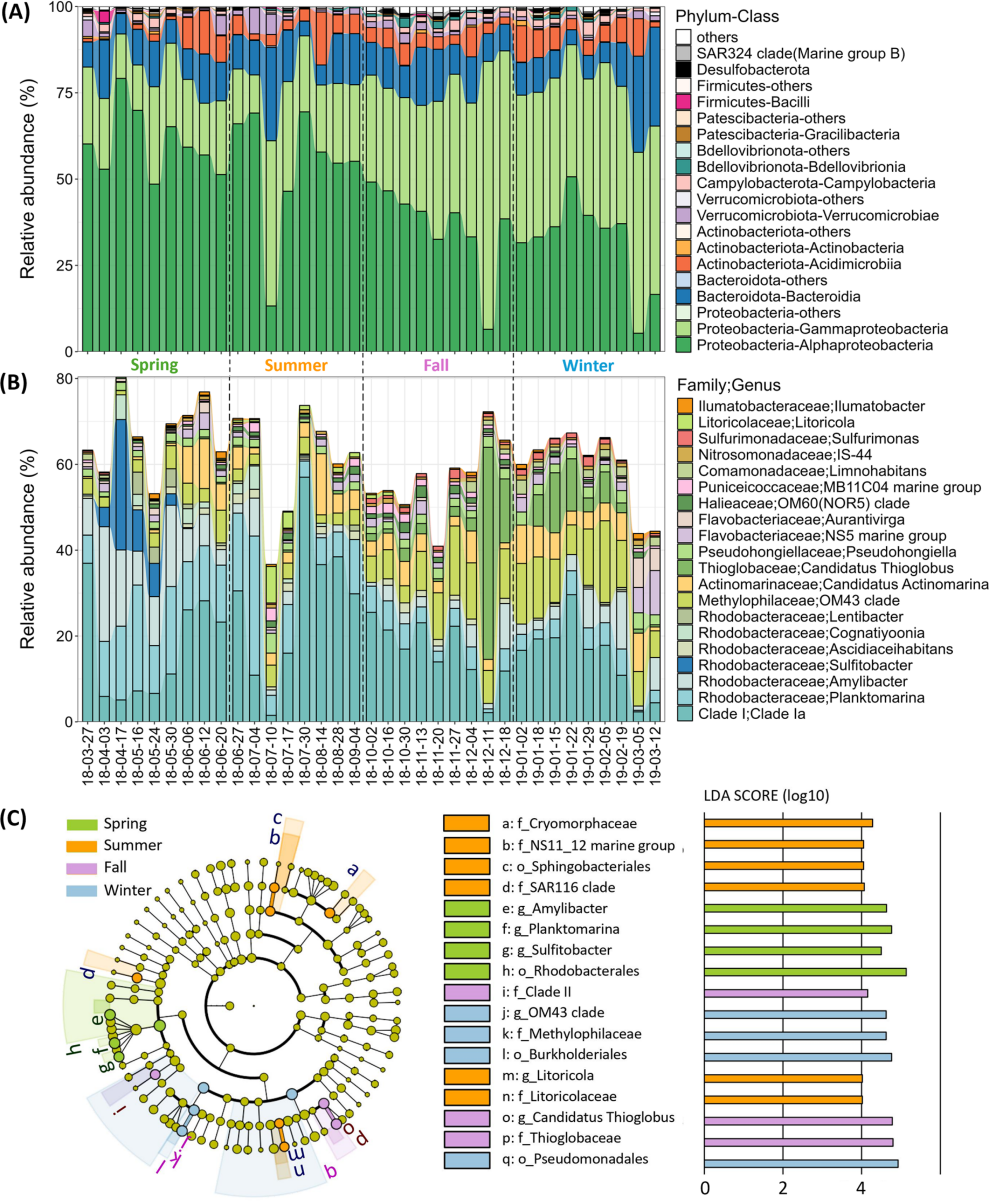
(A) Relative abundance of the 10 most abundant phyla and classes. (B) Relative abundance of the 20 most abundant genera and their corresponding families. (C) Linear discriminant analysis (LDA) effect size (LEfSe) indicating seasonally distinct biomarkers, with taxa from phylum to genus and an LDA score cutoff of 4.

Bacterial community composition among samples at different taxonomic levels was visualized using relative abundance bar plots (Figures 2, S2). Sequences were classified to the phylum, class, order, family, genus, and species levels with success rates of 100.0% ± 0.0%, 99.7% ± 0.4%, 99.5% ± 0.5%, 97.7% ± 1.8%, 70.0% ± 9.0%, and 57.7% ± 9.6%, respectively. Figure 2A displays the 10 most abundant phyla and classes. At the phylum level, *Proteobacteria* (77.8% ± 7.5%), *Bacteroidota* (11.8% ± 6.0%), and *Actinobacteriota* (5.3% ± 3.6%) were predominant across all samples. Seasonal trends were evident within the *Proteobacteria*: *Alphaproteobacteria* decreased from spring (60.4% ± 9.2%) to summer (54.0% ± 17.1%), then to fall (36.7% ± 11.8%), and winter (31.8% ± 12.5%). In contrast, *Gammaproteobacteria* increased from spring (19.7% ± 4.9%) to summer (24.2% ± 10.6%); then to fall (40.8% ± 14.3%), and winter (43.8% ± 4.6%). Seasonal variations, consistent with trends observed at the class level, were also noted at the order and family level, including *Rhodobacterales* and *Rhodobacteraceae* from *Alphaproteobacteria*, as well as *Pseudomonadales* and *Burkholderiales* from *Gammaproteobacteria* (Figure S2A, B). Figure 2B displays the 20 most abundant genera and their corresponding families. Within the *Rhodobacteraceae* family, *Plankomarina* (8.6% ± 6.9%) and *Amylibacter* (5.8% ± 5.9%) were particularly prevalent, with the highest relative abundance observed in spring (26.7 ± 9.3%) compared to summer (14.2% ± 11.4%), fall (7.8% ± 3.8%), and winter (9.1% ± 5.0%). *Sulfitobacter*, also within the *Rhodobacteraceae* family, was primarily present in spring, constituting 6.1% ± 9.2% of the community with a range from 0% to 30.4%. The *OM43 clade*, belonging to the *Methylophilaceae* family, showed a seasonal increase, starting at 3.8% ± 1.8% in spring and 3.3% ± 0.9% in summer, then rising to 9.5% ± 3.6% in fall and reaching 11.4%± 3.9% in winter. *Candidatus Thioglobus* from the *Thioglobaceae* family was mainly found in fall and winter, with relative abundances of 8.7% ± 15.0% and 5.3% ± 4.9%, respectively.

LDA effect size (LEfSe) was used to further investigate the difference in bacterial community composition across seasons. A bacterial taxon with a high LDA score in a given season might be a potential biomarker for that season. Taxa ranging from phylum to genus levels with LDA scores greater than 4 are shown in Figure 2C. The taxa with the most differential abundance in spring included order *Rhodobacterales*, and genera *Planktomarina*, *Amylibacter*, and *Sulfitobacter*. For summer, the most distinctive taxa were the families *Cryomorphaceae*, *SAR116 clade*, *NS11_12 marine group*, order *Sphingobacteriales*, as well as family *Litoricolaceae* and its genus *Litoricola*. In fall, family *Thioglobaceae* and its genus *Candidatus Thioglobus*, as well as family *Clade II* stand out, while in winter, the order *Burkholderiales*, family *Methylophilaceae* and its genus *OM43 clade*, as well as order *Pseudomonadales* were most abundantly different.

### High‐level bacterial phenotypes and predicted functions across seasons

**FIGURE 3:**
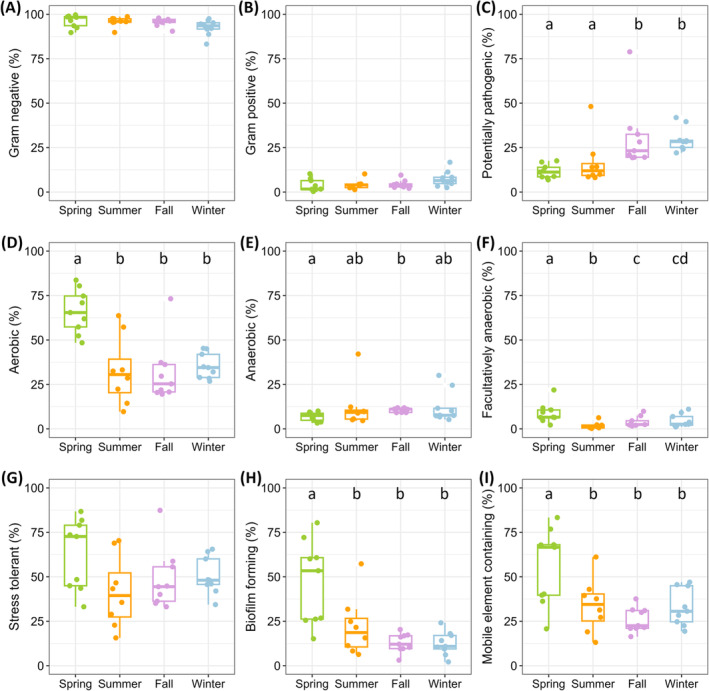
Relative abundance of bacterial phenotypes predicted by BugBase across seasons. **(**A) Gram negative, (B) Gram positive, (C) potentially pathogenic, (D) aerobic, (E) anaerobic, (F) facultatively anaerobic, (G) stress tolerant, (H) biofilm forming, and (I) mobile element containing. Differences among seasons were tested using the Kruskal–Wallis test, followed by the Wilcox test for post‐hoc comparison. Different lowercase letters indicate statistically significant differences at *p* <0.05.

The bacterial phenotypes of each sample, illustrating their physiological properties and potential resistance mechanisms, were predicted using BugBase. In the input OTU table aligned with the Greengenes v13_5 database, the OTUs recognized by BugBase and then used for phenotype predictions accounted for 94.47% ± 3.32% (*n* = 35, ranging from 81.8% to 98.6%) of all sequences in each sample. This high proportion indicates that the majority of sequences were included in the phenotype predictions. The relative abundance of each phenotype across different seasons was visualized using box plots (Figure 3). Gram‐negative bacteria (95.0% ± 3.5%) dominated all samples, compared to Gram‐positive bacteria (5.0% ± 3.5%) (Figure 3A, B). No significant seasonal differences in the relative abundance of either Gram‐negative or Gram‐positive bacteria were observed (Kruskal–Wallis, both *p* >0.05). Potentially pathogenic bacteria were more prevalent in fall (30.9% ± 17.9%) and winter (29.7% ± 0.06%) compared to spring (11.5% ± 3.7%) and summer (16.8% ± 12.5%) (Figure 3C, Wilcox, all *p* <0.05). No significant differences in potentially pathogenic bacteria between spring and summer or between fall and winter were found (Wilcox, both *p* >0.05). Regarding the types of respiration, it was found that aerobic bacteria were consistently abundant (41.7% ± 19.8%) with a peak in spring (66.1% ± 11.6%) (Figure 3D). Anaerobic bacteria (10.5% ± 7.4%) were most abundant in fall (10.7% ± 1.1%) and least abundant in spring (6.9% ± 2.3%) (Figure 3E). Facultatively anaerobic bacteria (4.8% ± 4.5%) exhibited the greatest abundance in spring (8.9% ± 5.4%), followed by winter (4.5% ± 3.4%) and fall (3.9 ± 2.7%), with the lowest abundance in summer (1.7 ± 1.9%) (Figure 3F). Stress‐tolerant bacteria showed no significant seasonal variations (Figure 3G, Kruskal–Wallis, *p* >0.05). Biofilm‐forming bacteria and mobile‐element‐containing bacteria were significantly more abundant in spring than in other seasons (Figure 3H, I, Wilcox, all *p* <0.05), with no significant differences observed between summer, fall, and winter (Wilcox, all *p* >0.05).

**FIGURE 4:**
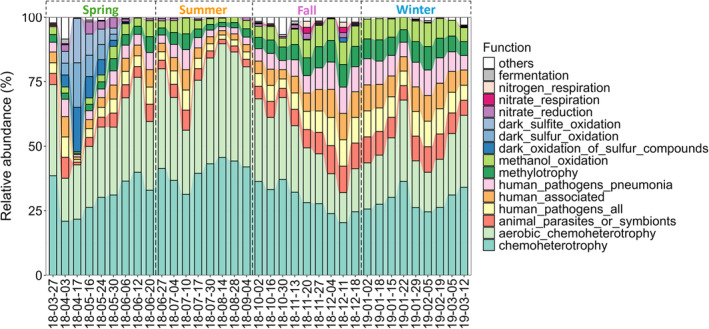
Relative abundance of the 15 most abundant potential bacterial functions, predicted by FAPROTAX, among samples.

Further analysis of bacterial functions was performed using the FAPROTAX algorithm, and the 15 most abundant potential bacterial functions among samples were visualized using the relative abundance bar plots (Figure 4). Among the 15 predicted functions, chemoheterotrophy (32.2% ± 6.9%), aerobic chemoheterotrophy (26.9% ± 8.5%), animal parasitism or symbiontism (6.2% ± 2.7%), human pathogenicity (6.2% ± 2.7%), human‐associated (6.2% ± 2.7%), human pathogenicity causing pneumonia (6.0% ± 2.7%), methylotrophy (5.2% ± 2.3%), and methanol oxidation (5.1% ± 2.4%) were predominant across all samples. Chemoheterotrophy and aerobic chemoheterotrophy were most abundant in summer, while the others predominated in fall and winter. In addition, functions related to sulphur oxidation, including dark sulphite oxidation (1.1% ± 3.3%, ranging from 0.0 to 17.2), dark sulphur oxidation (1.1% ± 3.3%, ranging from 0.0 to 17.2), and dark oxidation of sulphur compounds (1.2% ± 3.3%, ranging from 0.0 to 17.2), were predominantly or exclusively detected in spring.

### Effects of beach recreational activities and environmental factors on bacterial composition and function

**FIGURE 5:**
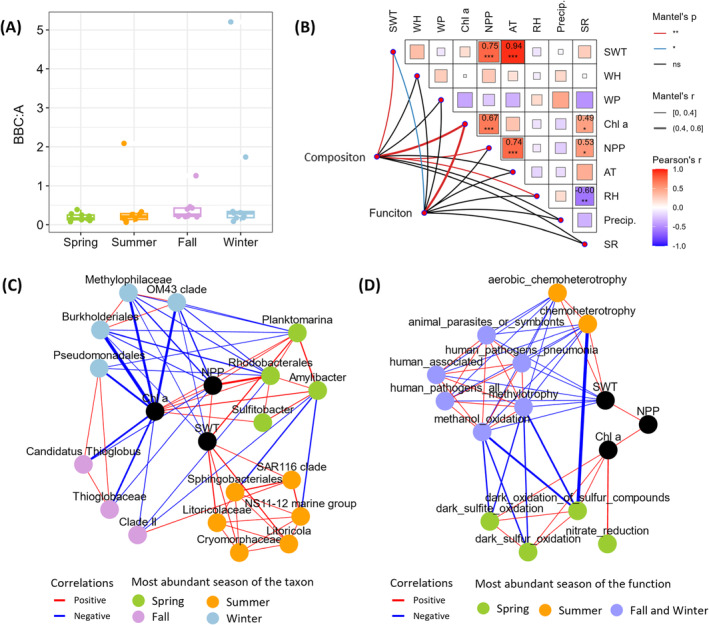
(A) The *Bacilli*, *Bacteroidota* and *Clostridia* to *Alphaproteobacteria* (BBC:A) ratio across seasons. (B) Pairwise comparisons of environmental variables shown at the upper‐right, with a colour gradient representing Pearson's correlation coefficient. Bacterial community composition and function were correlated to each environmental variable using partial Mantel tests (ns, not significant; *, *p* <0.05; **, *p* <0.01). SWT, seawater temperature; WH, wave height; WP, wave period; Chl *a*, chlorophyll *a*; NPP, net primary productivity; AT, air temperature; RH, relative humidity; Precip., precipitation; SR, solar radiation. Correlation networks of (C) bacterial composition (biomarkers) and (D) abundant functions with key environmental variables. Nodes representing bacterial taxa and functions are highlighted according to the seasons in which they are most abundant. Red lines denote significantly positive correlations, while blue lines indicate significantly negative correlations between adjacency parameters; the significance level was set at *p* <0.05. Line width represents the absolute value of Pearson's correlation coefficient, with only lines where *r* >0.4 displayed. Isolated nodes were discarded.

To assess the impact of beach recreational activities, particularly faecal inputs, we calculated the ratio of *Bacilli*, *Bacteroidota*, and *Clostridia* to *Alphaproteobacteria* (BBC:A) as an indicator. The BBC:A ratio, recognized as a proxy for estimating faecal matter contamination (Wu et al., 2010), has been applied in coastal seawater studies (Wang et al., 2019). In our study, the BBC:A ratio (0.50 ± 0.92) remained consistent across all seasons (Figure 5A), and was distinctly lower than the typical faecal ratios of 4–8 (Wu et al., 2010). We also checked the relative abundance of two traditional faecal indicator bacteria: *E. coli* and *Enterococcus*. *E. coli* was detected in only one spring sample with a relative abundance of 0.11%, while *Enterococcus* was not detected in any sample (Table S3.). Both the BBC:A ratio and the relative abundance of *E. coli* and *Enterococcus* indicate low faecal contamination levels in the studied surf zone seawater. To address the potential limitation of community‐targeted high‐throughput sequencing methods, which may miss low‐abundance organisms, we further reviewed water quality monitoring reports from 2018 and 2019 for coastal water at Ostend–Dunes and Seas (51°14′34′′N, 2°56′12′′E; approximately 360 m from our seawater collection site). These reports, conducted by the Flemish Environment Agency and the Department of Care (https://kwaliteitzwemwater.be), covered the period from 22 May 2018, to 10 September 2018, coinciding with late spring and summer of our sampling period when beach attendance peaks annually. The monitoring data showed *E. coli* levels at 48 ± 59 CFU/100 mL (*n* = 22, ranging from 5 to 216 CFU/100 mL), and *enterococci* levels at 14 ± 21 CFU/100 mL (*n* = 22, ranging from 1 to 108 CFU/100 mL) (Table S6). All these sampling days fell within the ‘Very good’ classification of coastal water quality according to the European Bathing Water Directive (2006/7/EC) (Standards for ‘Very Good’: Intestinal *enterococci* ≤200 CFU/100 mL, and *E. coli* ≤500 CFU/100 mL). This additional data further supports our conclusion of low faecal contamination levels in the study area, suggesting minimal impact from faecal inputs related to beach recreational activities. These findings reinforce that the study site was under ‘normal’ conditions—free from distinct pollution factors and disease outbreaks—during our sampling periods. However, it is important to acknowledge that other human activities, such as the introduction of skin‐associated bacteria and chemicals like sunscreen during swimming, could potentially influence bacterial communities in the surf zone. Since these factors were not the primary focus of our study due to their relatively minor significance in standard water quality assessments and the specific scope of our research objectives, we did not further analyze their potential impacts in this study.

To investigate the key environmental variables influencing bacterial communities, we analyzed correlations between bacterial composition and function with environmental variables (Figure 5B). The Mantel test revealed that four environmental variables—Chl *a*, SWT, NPP, and RH—significantly affected bacterial composition (all *p* <0.05). Two environmental variables, Chl *a* and SWT, significantly affected bacterial function (both *p* <0.05). NPP was significantly correlated with both Chl *a* and SWT (both *p* <0.05). To further explore the relationships between key environmental factors and specific bacterial taxa or functions, we constructed a correlation network using key environmental variables and biomarkers identified by LEfSe or the 15 most abundant functions predicted by FAPROTAX (Figure 5C, D). Most bacterial biomarkers for spring showed significant positive correlations with Chl *a* and NPP (all *p* <0.05), while those for summer exhibited significant positive correlations with SWT (all *p* <0.05). In contrast, biomarkers for fall were significantly negatively correlated with Chl *a* and NPP, and those for winter were significantly negatively correlated with Chl *a*, NPP, and SWT. Predicted functions abundant in spring significantly positively correlated with Chl *a*, those in summer with SWT, while those in fall and winter showed significant negative correlations with SWT (all *p* <0.05).

## DISCUSSION

This study investigated the structure and function of bacterial communities in the surf zone seawater of a recreational beach in Ostend, Belgium. The surf zone displayed distinct seasonal environmental heterogeneity: higher levels of Chl *a* in spring, and elevated SWT and AT during summer. NPP was also higher in both spring and summer, while these parameters gradually decreased through fall and reached their lowest levels in winter. Using full‐length 16S rRNA gene sequencing and the FAPROTAX algorithm, bacterial composition and potential functions were analyzed and then correlated with environmental variables to identify potential drivers. The results highlighted distinct seasonal differences in both bacterial composition and predicted functions, significantly influenced by SWT, Chl *a*, and NPP. Despite high levels of tourist activity during the summer, bacterial indicators of faecal matter contamination remained at very low levels compared to typical faecal‐contaminated environments (Wang et al., 2019; Wu et al., 2010), and were consistent across seasons. These findings suggest that bacterial communities in the surf zone seawater exhibit minimal faecal contamination related to beach recreational activities and are largely shaped by natural environmental cycles.

Higher richness and lower evenness were observed in spring (Figure 1A, B), likely due to the rapid succession of phytoplankton during this season. This process not only creates more niches for bacterial colonization, but also promotes the proliferation of certain bacterial taxa that thrive on the abundant resources provided by phytoplankton blooms (Camarena‐Gómez et al., 2018; Needham & Fuhrman, 2016). Conversely, lower richness and higher evenness were observed in fall and winter, suggesting a more balanced distribution of bacterial taxa during these cooler seasons (Figure 1A, B). This balance may result from a reduced resource availability, which limits the dominance of any single taxon and promotes a more uniform bacterial community (Sintes et al., 2013). The Shannon index was lowest in summer (Figure 1C), reflecting reduced richness and evenness (Figure 1A, B), possibly due to intensified competition for resources following the spring bloom and selective pressures from elevated temperatures.

Consistent with previous studies on surface seawater from various offshore and open sea environments (Sunagawa et al., 2015; Zhao et al., 2019), the bacteria in the surf zone seawater primarily comprised of Gram‐negative bacteria *Proteobacteria* (77.8% ± 7.5%) and *Bacteroidota* (11.8% ± 6.0%), and Gram‐positive bacteria *Actinobacteriota* (5.3% ± 3.6%) at the phylum level across all samples (Figure 2A). The bacterial community composition showed distinct seasonal variations from the class to species levels (Figure S3), and these seasonal changes were also reflected in their predicted physiological properties, resistance mechanisms, and metabolic functions (Figures 3 and 4). This variability underscores the dynamic nature of the surf zone environment, allowing for a detailed examination of how bacterial communities adapt across different seasons.

In spring, surf zone seawater displayed significant enrichment of the order *Rhodobacterales*, specifically genera including *Planktomarina*, *Amylibacter*, and *Sulfitobacter* from the family *Rhodobacteraceae* (Figures 2B, S2A). These genera contain aerobic anoxygenic phototrophs, which utilize bacteriochlorophyll *a* to enhance energy production in oxygen‐rich environments without producing oxygen as a byproduct of photosynthesis (Giebel et al., 2019; Oz et al., 2005). The dual metabolic strategy of these bacteria, combining phototrophy with aerobic oxidation of organic compounds, explains their significant positive correlations with Chl *a* and NPP (Figure 5C), and predicted higher aerobic phenotype by BugBase (Figure 3D). *Rhodobacteraceae* are early colonizers and therefore crucial in the development of biofilms in coastal seawater (Elifantz et al., 2013; Qian et al., 2022), which are hot spots of gene exchange mediated by the transfer of mobile elements (Abe et al., 2021). They also play an important role in thiosulfate oxidation (Ding et al., 2023). These known functions align with our predictions of higher biofilm‐forming and mobile‐element‐containing phenotypes (Figure 3H, I), as well as abundant dark sulphur/sulphite/sulphur compounds oxidation potential (Figure 4) in spring. Bacteria characterized with the potential of biofilm‐forming and mobile‐element‐containing were, however, not classified at the species level. This hampers our ability to assess their pathogenic potential or to elucidate associations with pathogens like *E. coli*, *Staphylococcus epidermidis*, and *Vibrio splendidus*, which were predominantly detected in spring and with which they could exchange genes (Table S3.). Enhanced molecular characterization is needed to better understand these bacteria's roles in coastal ecosystems and their impact on human health. *Sulfitobacter*, on the other hand, is the predominant cause of the dark sulphur/sulphite/sulphur compounds oxidation functions predicted by FAPROTAX based on the documented literature of cultivable strains (Table S4). Some species of *Sulfitobacter* possess the gene *dddL*, which encodes the enzyme catalyzing the conversion of dimethylsulfoniopropionate (DMSP) to gas dimethylsulfide (DMS) (Zeng et al., 2016). DMS serves as an info‐chemical, attracting animals for predation, and can contribute to cloud formation when released into the air (Zeng et al., 2016). Further investigation into the interactions between *Sulfitobacter* and phytoplankton (key producers of DMSP in the ocean), and *Sulfitobacter*'s role in the formation of ice nuclei and cloud condensation nuclei, could enhance our understanding of regional climate effects.

In summer, surf zone seawater was characterized by the prevalence of *Cryomorphaceae* (6.4% ± 3.6%), *SAR116 clade* (4.5% ± 1.0%), *NS11_12 marine group* (2.9% ± 2.4%), order *Sphingobacteriales* (2.9% ± 2.4%), as well as the family *Litoricolaceae* (2.0% ± 2.7%) and its genus *Litoricola* (2.0% ± 2.7%) (Figure 2C). Among them, *Cryomorphaceae*, particularly the uncultured bacterium with the subject ID AB974080.1.1430 (1.60% ± 0.98%, 5.19% ± 3.88%, 0.71% ± 1.26%, and 0.13% ± 0.35% in spring, summer, fall, and winter, respectively), was the most abundant and distinct in summer compared to other seasons. Most members of *Cryomorphaceae* are either strictly aerobic or facultatively anaerobic and rely on a chemoorganotrophic metabolism (Rosenberg et al., 2014), which may explain the predicted higher aerobic chemoheterotrophy function in summer (Figure 4). *Cryomorphaceae* has also been reported to be enriched in the sea surface microlayer (SML) of coastal seawater (Zäncker et al., 2018), a region known for its unique biochemical and physical properties that may enhance the survival and metabolic activities of certain microbes. Contrary to the findings by Zäncker et al. (2018), which reported a negative association of *Cryomorphaceae* with seawater temperature, our results revealed a significant positive correlation (Figure 5C). This discrepancy could be due to the adaptation of *Cryomorphaceae* bacterium AB974080.1.1430 to the SML environment, where enhanced sunlight exposure and nutrient interactions may mitigate the thermal stress typically detrimental at higher temperatures. Another explanation could involve differences in the properties of *Cryomorphaceae* at the genus or species level. In this study, another highly prevalent *Cryomorphaceae* bacterium—uncultured *Sphingobacterium* sp. EB080_L08E11 (subject ID GU474939.107055.108550) was predominantly detected in fall (1.76% ± 1.32%) and winter (2.02% ± 1.33%) compared to spring (0.67% ± 1.03%) and summer (0.36% ± 0.94%). At present, little is known about either species.

From mid‐ or late fall, family *Thioglobaceae* and its genus *Candidatus Thioglobus*, order *Burkholderiales*, family *Methylophilaceae*, and its genus *OM43 clade*, as well as order *Pseudomonadales*, became abundant and maintained their prevalence throughout the winter (Figures 2, S2). The family *Thioglobaceae* and its genus *Candidatus Thioglobus* were identified as biomarkers for fall, whereas the others were identified as biomarkers for winter (Figure 2C). *Thioglobaceae* and *Candidatus Thioglobus* were classified as fall biomarkers, probably because of their significant abundance in the last two samples of fall (Figure 2B). This may be related to the death of marine phytoplankton from mid‐fall onwards due to dropping temperatures, with the resulting organic matter being decomposed by anaerobic bacteria producing hydrogen sulphide (H_2_S). *Thioglobaceae* has been reported to be particularly abundant and active in oxygen minimum zones, playing a pivotal role in oxidizing H_2_S into sulphate (Meier et al., 2017), consistent with our prediction of higher phenotype of anaerobic bacteria in fall (Figure 3E), and the significant negative correlation between *Thioglobaceae* and Chl *a* (Figure 5C). Biomarkers for winter showed significant negative correlations with Chl *a*, net primary productivity, and SWT (Figure 5C), suggesting that these bacteria thrive under the cooler and less biologically active conditions typical of winter. This is likely because they have evolved adaptations that enable efficient utilization of limited resources (e.g., dissolved organic matter, methane, and H_2_S from decomposed plant and animal remains) in environments with reduced temperatures and decreased competition. Among them, *Methylophilaceae*, known for methylotrophs, exhibited elevated levels of methylotrophy and methanol oxidation functions, as predicted by FAPROTAX (Figure 4). The predicted functions of methylotrophy and methanol oxidation showed a significant negative correlation with Chl *a*, further supporting the utilization of limited resources from the decomposition of organic matter, like phytoplankton, during the winter.

Fall and winter showed higher levels of potentially pathogenic phenotype and predicted health‐relevant functions compared to spring and summer (Figures 3C and 4). The higher levels of potentially pathogenic phenotype mainly stem from the higher relative abundance of *Methylophilaceae* and *SUP05 cluster*, predicted by BugBase based on phylogenetic relationships (Figure S4). To our knowledge, no studies have explicitly reported the pathogenicity of *Methylophilaceae* and *SUP05 cluster*. Nevertheless, recent studies have highlighted environmental interactions involving *Methylophilaceae* and the *SUP05 cluster* that suggest their potential for pathogenic behaviour. For instance, Shen et al. (2019) observed an increased abundance of *Methylophilaceae* following exposure to pharmaceuticals (particularly tylosin) in soil and plants. Hultman et al. (2018) discovered that *Methylophilaceae*, lacking the tetracycline resistance gene *tetM*, acquired *tetM* post‐wastewater treatment. Furthermore, the *SUP05 cluster*, active in oxygen minimum zones, often exhibits infections with multiple viral contigs, suggesting a high propensity for horizontal gene transfer (HGT), which could facilitate pathogenic potential (Roux et al., 2014). According to predictions by FAPROTAX, the most abundant taxon associated with human health is an unclassified bacterium from the *OM43 clade* (subject ID FJ545543.1.1535) within the *Methylophilaceae* family, which dominated at 74.1% ± 20.3% among these abundant health‐relevant taxa (Table S5). This aligns with the result from BugBase, suggesting that the potentially high pathogenic risks might be due to these taxa containing antibiotics resistance genes (ARGs). However, whether these potential ARG carriers are harmful pathogens or simply harmless constituents of the environmental microbiota remains further investigation.

No significant differences were found in the relative abundance of common faecal indicators, including *E. coli* and *Enterococcus*, and the BBC:A ratio, between summer, when tourism peaks, and other seasons (Table S3., Figure 5A). This indicates that the impact of summer recreational activities on faecal contamination in the studied surf zone seawater is minimal. This was further confirmed by the low levels of *E. coli* and *enterococci* in summer based on culturable methods reported by the Flemish Environment Agency and the Department of Care, which fall within the ‘Very good’ classification of coastal water quality according to the European Bathing Water Directive (2006/7/EC) (Table S6). Şanlıtürk and Güran (2022) also reported no significant differences in percentage detection frequencies of *E. coli* across beaches with different levels of recreational use. Seasonal variations in environmental factors, including Chl *a*, net primary productivity, and seawater temperature, were identified as the main drivers for bacterial community composition and function in this study. Further explanations mentioned in the above discussions involved oxygen and nutrient utilization. For clearer understanding of the mechanisms driving these bacterial changes across different seasons, further studies that include measurements of these relevant environmental parameters are recommended.

## CONCLUSION

This study of bacterial communities in the surf zone seawater of a recreational beach in Ostend, Belgium—an area without a history of severe pollution or disease outbreaks—revealed that these communities were significantly influenced by seasonal variations in environmental factors including Chl *a*, net primary productivity, and seawater temperature, with minimal impact of faecal inputs from beach recreational activities. The adaptability of different bacterial populations to different environmental conditions not only underscores their role in maintaining ecological balance but also highlights their contribution to nutrient cycling within the marine ecosystem. This function is crucial for sustaining ecosystem health, which in turn potentially enhances public health by maintaining clean and safe water conditions. Bacteria, particularly those that exhibited higher biofilm‐forming potential in spring and enriched under low oxygen conditions in winter, may exhibit higher levels of antibiotic resistance. Further investigation into antibiotic resistance genes within these populations is essential to assess their potential impact on public health and to enhance our understanding of microbial resistance mechanisms in surf zone environments.

## AUTHOR CONTRIBUTIONS

**Yunmeng Li:** Conceptualization; formal analysis; visualization; writing – original draft; writing – review and editing. **Pascal I. Hablützel:** Formal analysis; writing – review and editing. **Zixia Liu:** Conceptualization; investigation; writing – review and editing. **Emmanuel Van Acker:** Investigation; writing – review and editing. **Colin R. Janssen:** Conceptualization; writing – review and editing. **Jana Asselman:** Conceptualization; writing – review and editing; supervision. **Maarten De Rijcke:** Conceptualization; investigation; funding acquisition; writing – original draft; writing – review and editing; supervision.

## CONFLICT OF INTEREST STATEMENT

The authors declare no conflict of interest.

## Supporting information


**Data S1:** Supporting Information

**Table S3.** Relative abundance of identified taxa at the species level.
**Table S4.** Abundant functions associated with human health and sulphur or sulphite oxidation, and their respective taxa predicted by FAPROTAX.
**Table S5.** Relative abundance of taxa associated with abundant health‐relevant functions predicted by FAPROTAX.

## Data Availability

The data and code that support the findings of this study are openly available in the online repository Marine Data Archive, https://doi.org/10.14284/669.
