# Supplementary material for: Seasonal dynamics of bacterial community structure and function in the surf zone seawater of a recreational beach in Ostend, Belgium
Source: Environ Microbiol Rep. 2024 Nov 8;16(6):e70031. doi: 10.1111/1758-2229.70031 (PMC11544449; doi:10.1111/1758-2229.70031)
Supplement: Supplementary file 1 — Data S1: Supporting Information [file EMI4-16-e70031-s001.docx]

**aSUPPLEMENTARY MATERIALS**

**Seasonal dynamics of bacterial community structure and function in surf zone seawater of a recreational beach in Ostend, Belgium**

Yunmeng Li ^1,2^, Pascal I. Hablützel ^1,3^, Zixia Liu ^2^, Emmanuel Van Acker ^4,^^[[1]](#footnote-2)^, Colin R. Janssen ^2,4^, Jana Asselman ^2^, Maarten De Rijcke ^1^

^1^Flanders Marine Institute (VLIZ), InnovOcean Campus, Jacobsenstraat 1, 8400 Ostend, Belgium

^2^Blue Growth Research Lab, Ghent University, Wetenschapspark 1, 8400 Ostend, Belgium

^3^Department of Biology, Vrije Universiteit Brussel, Pleinlaan 2, 1050 Brussels, Belgium

^4^Laboratory of Environmental Toxicology and Aquatic Ecology (GhEnToxLab), Department of Animal Sciences and Aquatic Ecology, Ghent University, Coupure Links 653, 9000 Ghent, Belgium

**Correspondence**

Maarten De Rijcke, Flanders Marine Institute (VLIZ), InnovOcean Campus, Jacobsenstraat 1, 8400 Ostend, Belgium. Email: [maarten.de.rijcke@vliz.be](mailto:maarten.de.rijcke@vliz.be).

**Contents**

- **Table S1** Sample descriptions.
- **Figure S1** Successfully sequenced samples indicated by rarefaction curves based on observed OTUs, and their representation of seasonal environmental variability.
- **Table S2** Average environmental conditions during sampling among different seasons of the year.
- **Figure S2** Relative abundance of the 20 most abundant orders and families.
- **Table S3** Relative abundance of identified taxa at the species level. => see additional excel file.
- **Figure S3** Differences of bacterial community structure across seasons at different taxonomic levels.
- **Table S4** Abundant functions associated with human health and sulfur or sulfite oxidation, and their respective taxa predicted by FAPROTAX.
- **Figure S4** Relative abundance of taxa categorized as potentially pathogenic phenotype by BugBase.
- **Table S5** Relative abundance of taxa associated with abundant health-relevant functions predicted by FAPROTAX. => see additional excel file.
- **Table S6** Water quality monitoring for coastal water at Oostende – Dunes and Seas (51°14′34′′N, 2°56′12′′E; approximately 360 m from our seawater collection site) conducted by the Flemish Environment Agency and the Department of Care from March 2018 to March 2019.

**Table S1** Weekly sampling events of the surf zone seawater in a recreational sandy beach in Ostend, Belgium over a year, environmental variables, and the final count of sequences by Nanopore full-length 16S rRNA gene sequencing used for subsequent analysis.

- All dates and times are given in Belgian local time. Seasons were defined based on astronomical equinoxes and solstices for the years 2018 and 2019. Specifically, spring was defined as March 20, 2018 to June 20, 2018; summer as June 21, 2018 to September 22, 2018; fall as September 23, 2018 to December 20, 2018; and winter as December 21, 2018 to March 19, 2019.
- SWT, seawater temperature; WH, wave height; WP, wave period; Chl *a*, chlorophyll *a*; NPP, net primary productivity; AT, air temperature; SR, solar radiation; RH, relative humidity; Precip., precipitation.
- NA, not available. *, no FASTQ files or OTU tables generated. ‘Yes’ indicates samples that generated sequences with rarefaction curves reaching a plateau.

| No. | Date | Time | Season | SWT | WH | WP | Chl *a* | NPP | AT | SR | RH | Precip. | Sequencing batch | Final sequence counts | Passed sequencing |
| --- | --- | --- | --- | --- | --- | --- | --- | --- | --- | --- | --- | --- | --- | --- | --- |
|  |  |  |  | (℃) | (cm) | (s) | (mg/m^3^) | (mg C/m^2^/d) | (℃) | (W/m^2^) | (%) | (mm) |  |  |  |
| 1 | 18-03-27 | 12:00 | Spring | 5.2 | 24 | 4.38 | 4.62 | 1,821.18 | 4.8 | 97.8 | 89.37 | 0.1 | 1 | 81,835 | Yes |
| 2 | 18-04-03 | 13:00 | Spring | 7.4 | 44 | 3.21 | 11.41 | 3,693.57 | 10.7 | 400.4 | 84.53 | 0 | 1 | 12,945 | Yes |
| 3 | 18-04-10 | 11:15 | Spring | 9 | 55 | 5 | 0.78 | 672.83 | 10.2 | 132 | 92.26 | 0 | 1 | NA* | No |
| 4 | 18-04-17 | 14:15 | Spring | 10.3 | 27 | 2.37 | 17.51 | 5,415.63 | 13.7 | 569.8 | 69.39 | 0 | 1 | 52,521 | Yes |
| 5 | 18-04-25 | 15:30 | Spring | 13.3 | 81 | 3.58 | 17.53 | 7,798.03 | 11.9 | 573.2 | 71.44 | 0.1 | 1 | NA* | No |
| 6 | 18-05-02 | 16:00 | Spring | 11.6 | 42 | 3.54 | 12.69 | 7,136.71 | 13.6 | 691.7 | 54.51 | 0 | 1 | NA* | No |
| 7 | 18-05-09 | 16:00 | Spring | 14.6 | 39 | 3.1 | 15.43 | 9,264.86 | 13.3 | 733.4 | 84.26 | 0 | 1 | NA* | No |
| 8 | 18-05-16 | 11:30 | Spring | 14 | 110 | 3.54 | 15.43 | 9,264.86 | 13.3 | 327.5 | 85.96 | 0.1 | 1 | 73,412 | Yes |
| 9 | 18-05-24 | 13:30 | Spring | 16.2 | 66 | 4.08 | 7.64 | 6,296.72 | 17.5 | 575.6 | 74.66 | 0 | 1 | 36,280 | Yes |
| 10 | 18-05-30 | 12:00 | Spring | 17.7 | 49 | 4.31 | 13.79 | 10,573.05 | 16.3 | 215.7 | 90.83 | 0 | 1 | 19,194 | Yes |
| 11 | 18-06-06 | 11:15 | Spring | 16.8 | 91 | 3.62 | 8.19 | 7,067.75 | 14.7 | 263.6 | 88.38 | 0 | 1 | 53,275 | Yes |
| 12 | 18-06-12 | 11:00 | Spring | 17.5 | 83 | 3.46 | 5.47 | 6,398.15 | 15.2 | 263.3 | 84.47 | 0 | 1 | 48,147 | Yes |
| 13 | 18-06-20 | 14:30 | Spring | 18 | 60 | 3.53 | 12.02 | 11,040.47 | 18.7 | 560.4 | 82.2 | 0 | 1 | 41,854 | Yes |
| 14 | 18-06-27 | 16:00 | Summer | 18.8 | 96 | 3.86 | 9.2 | 9,518 | 18.4 | 883.4 | 68.94 | 0 | 1 | 69,491 | Yes |
| 15 | 18-07-04 | 11:00 | Summer | 19.7 | 69 | 3.66 | 8.25 | 8,799 | 17.3 | 300.9 | 79.39 | 0 | 1 | 57,295 | Yes |
| 16 | 18-07-10 | 14:30 | Summer | 20.6 | 154 | 4.49 | 8.25 | 8,799 | 16.3 | 463.2 | 71.08 | 0.1 | 6 | 9,568 | Yes |
| 17 | 18-07-17 | 11:45 | Summer | 21 | 83 | 3.65 | 6.38 | 7,395.08 | 19.2 | 425.7 | 75.18 | 0 | 1 | 44,850 | Yes |
| 18 | 18-07-24 | 11:00 | Summer | 22.2 | 24 | 2.74 | 7.99 | 8,133.07 | 20.8 | 276.9 | 73.07 | 0 | 1 | NA* | No |
| 19 | 18-07-30 | 15:30 | Summer | 22.2 | 32 | 2.63 | 6.12 | 6,548.48 | 23.7 | 420.9 | 71.09 | 0 | 1 | 55,042 | Yes |
| 20 | 18-08-07 | 11:30 | Summer | 23.3 | 19 | 3.45 | 6.06 | 6,209.21 | 22.9 | 320 | 71.21 | 0 | 4 | NA* | No |
| 21 | 18-08-14 | 10:30 | Summer | 20.8 | 63 | 3.4 | 5.2 | 5,673.98 | 18.8 | 120.7 | 89.29 | 0.4 | 2 | 5,027 | Yes |
| 22 | 18-08-20 | 14:30 | Summer | 20.6 | 31 | 3.29 | 5.2 | 5,673.98 | 20.2 | 196 | 84.22 | 0 | 4 | NA* | No |
| 23 | 18-08-28 | 14:00 | Summer | 19 | 34 | 3.79 | 4.15 | 4,666.09 | 18.2 | 390.7 | 77.49 | 0 | 4 | 5,946 | Yes |
| 24 | 18-09-04 | 11:00 | Summer | 19 | 90 | 3.53 | 4.79 | 4,865.86 | 18.4 | 31.9 | 92.72 | 0.4 | 4 | 7,266 | Yes |
| 25 | 18-09-11 | 11:00 | Summer | 18.3 | 90 | 4.16 | 5.22 | 4,984.35 | 17.7 | 100.9 | 86.39 | 0 | 4 | 80 | No |
| 26 | 18-09-19 | 11:00 | Summer | 18 | 33 | 2.92 | 5.83 | 5,076.14 | 17 | 91.4 | 88.58 | 0 | 4 | 13 | No |
| 27 | 18-09-25 | 10:00 | Fall | 16.1 | 60 | 5.04 | 4.26 | 3,698.63 | 11.1 | 2 | 73.79 | 0 | 4 | 28 | No |
| 28 | 18-10-02 | 14:00 | Fall | 15.2 | 114 | 4.15 | 4.08 | 3,400.13 | 14.7 | 115.9 | 86.66 | 0.2 | 2 | 6,244 | Yes |
| 29 | 18-10-09 | 10:15 | Fall | 15 | 31 | 3.24 | 5.76 | 3,705.03 | 10.9 | 0 | 91.07 | 0 | 4 | 122 | No |
| 30 | 18-10-16 | 11:00 | Fall | 16 | 55 | 5.76 | 5.16 | 3,379.3 | 14.5 | 15.6 | 80.58 | 0 | 2 | 6,579 | Yes |
| 31 | 18-10-23 | 11:00 | Fall | 15.1 | 171 | 4.57 | 5.16 | 3,379.3 | 11.6 | 6.9 | 79.09 | 0 | 6 | NA* | No |
| 32 | 18-10-30 | 10:30 | Fall | 11.8 | 92 | 5.66 | 4.07 | 1,872.78 | 8.7 | 9.8 | 84.59 | 3.1 | 3 | 11,767 | Yes |
| 33 | 18-11-05 | 15:00 | Fall | 11 | 25 | 3.5 | 4.8 | 2,063.42 | 12.9 | 317.7 | 81.44 | 0 | 4 | 67 | No |
| 34 | 18-11-13 | 11:00 | Fall | 11.1 | 70 | 3.8 | 4.54 | 1,662.82 | 11.3 | 110.5 | 83.46 | 0 | 5 | 5,103 | Yes |
| 35 | 18-11-20 | 10:00 | Fall | 8.8 | 157 | 5.13 | NA | NA | 4 | 6.6 | 76.26 | 0 | 3 | 4,994 | Yes |
| 36 | 18-11-27 | 14:00 | Fall | 8 | 33 | 4.73 | NA | NA | 6.5 | 100.6 | 86.44 | 0 | 3 | 12,657 | Yes |
| 37 | 18-12-04 | 11:00 | Fall | 9.6 | 91 | 4.74 | NA | NA | 8.2 | 51.9 | 80.25 | 0 | 3 | 10,977 | Yes |
| 38 | 18-12-11 | 14:00 | Fall | 9 | 92 | 5.34 | NA | NA | 8.1 | 190 | 64.46 | 0 | 6 | 36,236 | Yes |
| 39 | 18-12-18 | 11:00 | Fall | 6.9 | 29 | 3.82 | NA | NA | 7 | 33.2 | 94 | 0 | 3 | 10,946 | Yes |
| 40 | 18-12-24 | 10:00 | Winter | 9 | 70 | 3.65 | NA | NA | 8.5 | 0 | 83.7 | 0 | 3 | 3,703 | No |
| 41 | 19-01-02 | 11:00 | Winter | 7.1 | 203 | 5.87 | NA | NA | 7.3 | 0 | 65.21 | 0 | 3 | 5,057 | Yes |
| 42 | 19-01-08 | 10:30 | Winter | 6.6 | 256 | 5.61 | NA | NA | 7.9 | 16.4 | 77.46 | 0 | 3 | 11,440 | Yes |
| 43 | 19-01-15 | 14:00 | Winter | 6.7 | 90 | 4.07 | NA | NA | 7.3 | 210.3 | 85.92 | 0 | 3 | 19,130 | Yes |
| 44 | 19-01-22 | 13:30 | Winter | 5 | 44 | 3.54 | NA | NA | 2.8 | 66.8 | 89.86 | 0.2 | 3 | 5,369 | Yes |
| 45 | 19-01-29 | 11:00 | Winter | 5.2 | 59 | 5.14 | 2.33 | 631.83 | 1.2 | 69.2 | 84.02 | 0 | 3 | 29,937 | Yes |
| 46 | 19-02-05 | 14:00 | Winter | 4.6 | 37 | 3.47 | 3.4 | 894.16 | 5.8 | 202.5 | 90.08 | 0 | 2 | 27,124 | Yes |
| 47 | 19-02-12 | 11:00 | Winter | 6.6 | 54 | 5.11 | 3.01 | 1,163.12 | 5.2 | 91.2 | 88.15 | 0 | 3 | 27 | No |
| 48 | 19-02-19 | 14:00 | Winter | 7 | 56 | 3.26 | 4.46 | 1,541.19 | 8.6 | 414.4 | 79.75 | 0 | 2 | 21,036 | Yes |
| 49 | 19-02-26 | 11:00 | Winter | 7.3 | 16 | 3.7 | 4.65 | 1,888.47 | 7.7 | 209.9 | 82.45 | 0 | 6 | NA* | No |
| 50 | 19-03-05 | 14:00 | Winter | 8.7 | 77 | 3.63 | 4.65 | 1,888.47 | 8.8 | 474.1 | 69.36 | 0 | 7 | 6,862 | Yes |
| 51 | 19-03-12 | 9:30 | Winter | 8.2 | 77 | 3.85 | 4.92 | 1,983.05 | 7.2 | 22.4 | 68.29 | 0 | 6 | 18,994 | Yes |
| 52 | 19-03-19 | 14:00 | Winter | 9 | 64 | 6.09 | 4.98 | 2,297.41 | 9.4 | 530.2 | 77.68 | 0 | NA | NA* | No |


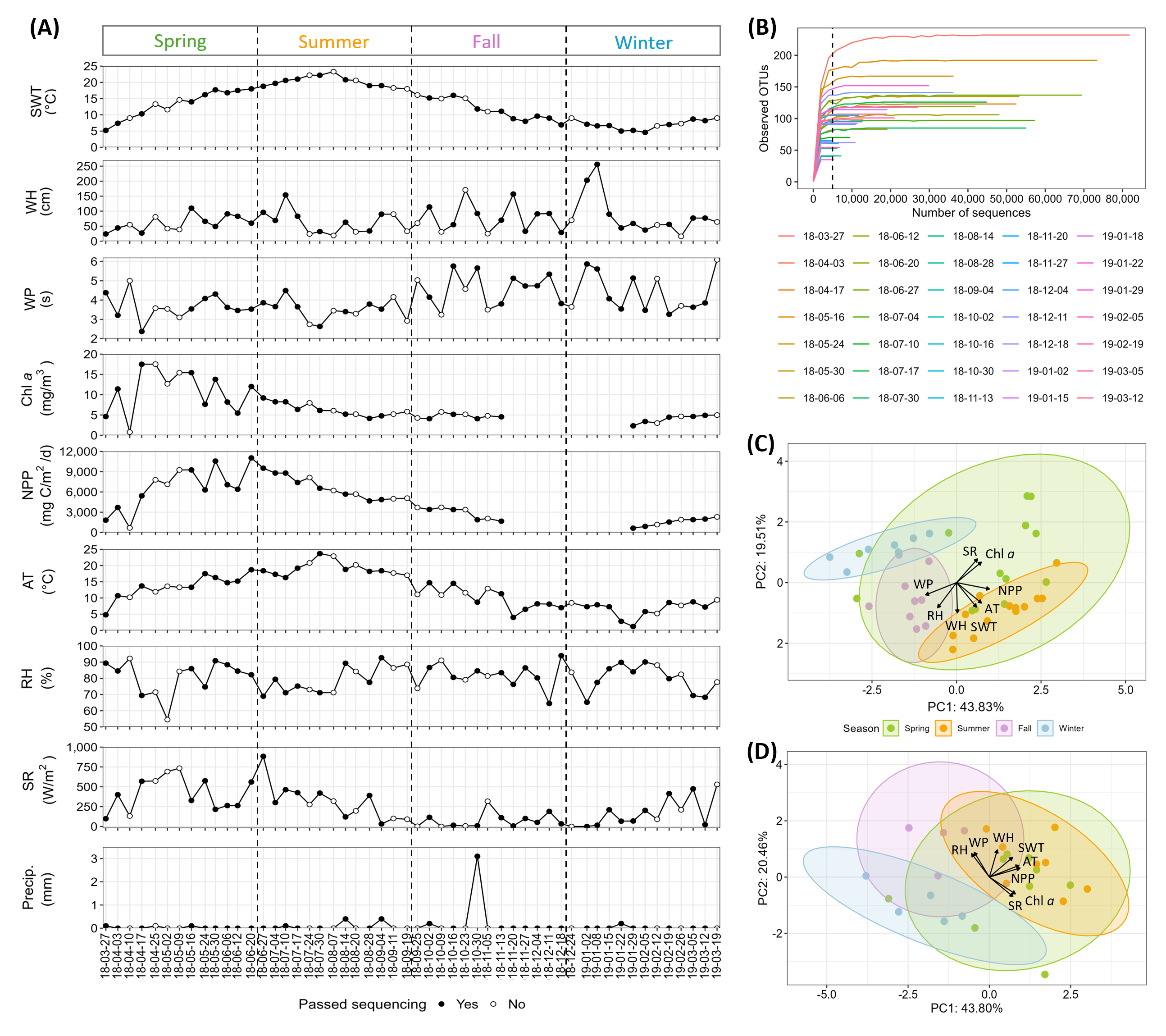


**Figure S1** **(A)** Temporal fluctuation of environmental variables across different seasons with sample points marked to indicate whether they passed sequencing criteria (Yes; 35 samples) or not (No; 17 samples). SWT, seawater temperature; WH, wave height; WP, wave period; Chl *a*, chlorophyll *a*; NPP, net primary productivity; AT, air temperature; RH, relative humidity; SR, solar radiation; Precip., Precipitation. **(B)** Rarefaction curves for the 35 samples that passed sequencing. The dotted line indicates the least number (4,994) of sequences. **(C)** Principal component analysis (PCA) ordination plots of environmental variables from the complete set (52 samples), with 42 included due to the unavailability of Chl *a* and NPP data in 10 samples. **(D)** PCA ordination plots of environmental variables from the successfully sequenced 35 samples, with 26 included due to the unavailability of Chl *a* and NPP data in 9 samples.

**Table S2** Average environmental conditions during sampling among different seasons of the year (Mean ± Standard deviation (SD)).

| Variables | Spring (*n* = 9) | Summer (*n* = 8) | Fall (*n* = 9) | Winter (*n* = 9) |
| --- | --- | --- | --- | --- |
| SWT (℃) | 13.7±4.6^a^ | 20.1±1.1^b^ | 10.7±3.0^ac^ | 6.6±1.3^d^ |
| WH (cm) | 61.6±27.4 | 77.6±36.4 | 81.4±38.0 | 99.9±72.1 |
| WP (s) | 3.6±0.6^a^ | 3.6±0.5^a^ | 4.8±0.7^b^ | 4.3±0.9^ab^ |
| AT (℃) | 13.9±3.9^a^ | 18.8±2.0^b^ | 9.2±3.4^c^ | 6.3±2.5^c^ |
| SR (W/m^2^) | 363.8±163.8^a^ | 379.7±239.5^a^ | 70.5±59.0^b^ | 164.0±166.6^ab^ |
| RH (%) | 83.3±6.7 | 78.1±8.2 | 81.9±7.7 | 78.9±8.9 |
| Precip. (mm) | 0.0±0.0 | 0.1±0.2 | 0.4±1.0 | 0.0±0.1 |

The superscripts present the results of Wilcox test, and the data with different superscripts in the same row are significantly different (*p* < 0.05).

SWT, seawater temperature; WH, wave height; WP, wave period; AT, air temperature; SR, solar radiation; RH, relative humidity; Precip., precipitation.


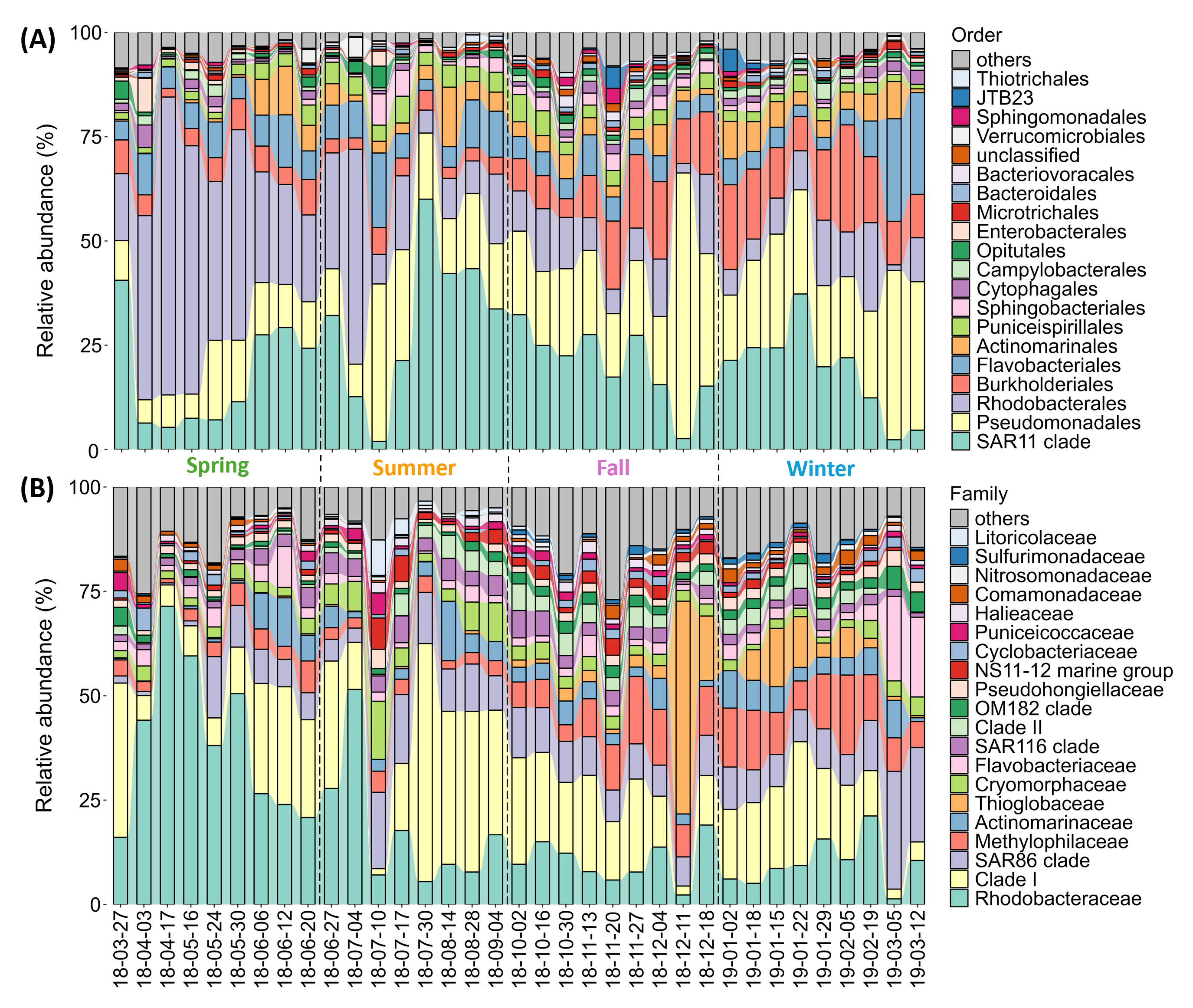


**Figure S2** Relative abundance of the 20 most abundant **(A)** orders and **(B)** families.


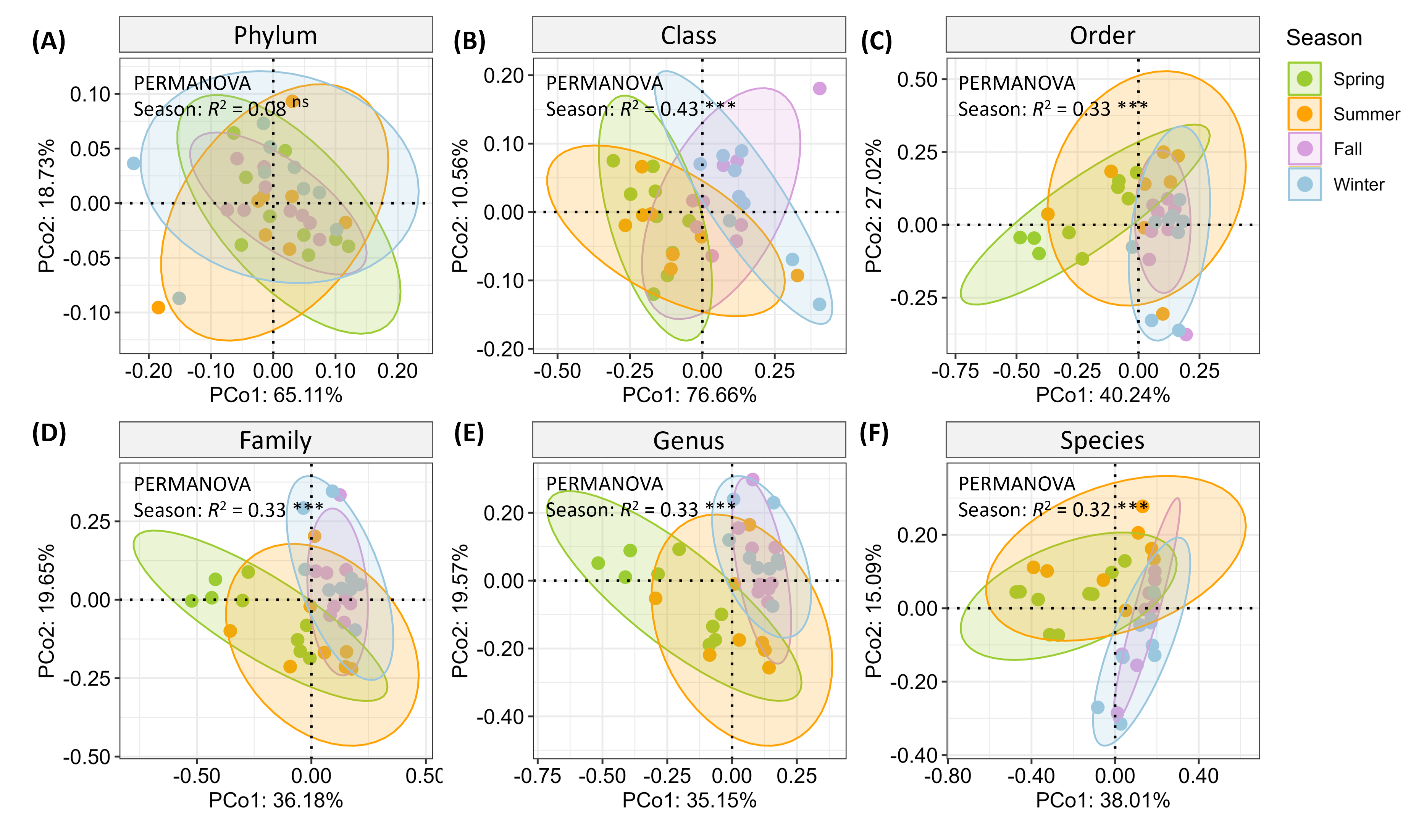


**Figure S3** Principal coordinate analyses (PCoA) and PERMANOVA tests of bacterial communities among different seasons based on Bray-Curits distances using the relative abundance of phylum **(A)**, class **(B)**, order **(C)**, family **(D)**, genus **(E)**, and species **(F)**. ns, not significant; ***, *p* < 0.001.

**Table S4** Abundant functions associated with human health and sulfur or sulfite oxidation, and their respective taxa predicted by FAPROTAX.

| Functions | | | Bacterial taxa | | |
| --- | --- | --- | --- | --- | --- |
| animal_parasites_or_symbionts | human_pathogens_all/human_associated |  | f__*Propionibacteriaceae* | g__*Cutibacterium* | |
|  |  |  | f__*Arcobacteraceae* | g__*Arcobacter* | s__*Cutibacterium acnes* |
|  |  |  | f__*Bacillaceae* | g__*Bacillus* | s__*Arcobacter butzleri 7h1h* |
|  |  |  |  |  | s__*Arcobacter cryaerophilus ATCC 43158* |
|  |  |  | f__*Staphylococcaceae* | g__*Staphylococcus* | s__*Streptococcus pneumoniae* |
|  |  |  | f__*Peptostreptococcaceae* | g__*Clostridioides* | s__*Streptococcus mitis* |
|  |  |  |  |  | s__*Staphylococcus epidermidis* |
|  |  |  |  |  | s__*Staphylococcus epidermidis RP62A phage SP-beta* |
|  |  |  | f__*Xanthobacteraceae* | g__*Afipia* | s__*Clostridioides difficile* |
|  |  | human_pathogens_pneumonia | f__*Alcaligenaceae* | g__*GKS98 freshwater group* |  |
|  |  |  | f__*Comamonadaceae* | g__*Aquabacterium* |  |
|  |  |  |  | g__*Candidatus Symbiobacter* |  |
|  |  |  |  | g__*Limnohabitans* |  |
|  |  |  |  | g__*Polaromonas* |  |
|  |  |  |  | g__*Rhodoferax* |  |
|  |  |  |  | g__*RS62 marine grou* | s__*Betaproteobacteria bacterium MOLA814* |
|  |  |  | f__*Methylophilaceae* | g__*Candidatus Methylopumilus* |  |
|  |  |  |  | g__*OM43 clade* |  |
|  |  |  | f__*Nitrosomonadaceae* | g__*DSSD61* |  |
|  |  |  |  | g__*IS-44* |  |
|  |  |  |  | g__*Nitrosomonas* |  |
|  |  |  | f__*Oxalobacteraceae* | g__*Massilia* |  |
|  |  |  | f__*Coxiellaceae* | g__*Coxiella* |  |
|  |  |  | f__*Enterobacteriaceae* | g__*Escherichia-Shigella* | s__*Escherichia coli* |
|  |  |  | f__*Pasteurellaceae* | g__*Haemophilus* | s__*Haemophilus parainfluenzae* |
|  |  |  | f__*Vibrionaceae* | g__*Vibrio* | s__*Vibrio tapetis* |
|  |  |  | f__*Francisellaceae* | g__*Francisella* | s__*Francisella sp. FSC1006* |
|  |  |  |  |  | s__*Francisella sp. TX077308* |
| dark sulfur oxidation  dark_oxidation_of_sulfur_compounds | | dark sulfite oxidation | f__*Rhodobacteraceae* | g__*Sulfitobacter* | s__*Sulfitobacter sp. KMM 6006* |
|  |  |  | f__*Thioglobaceae* | g__*SUP05 cluster* | s__*Candidatus Thioglobus singularis PS1* |
|  |  |  |  |  | s__*gamma proteobacterium SCGC AAA076-D02* |


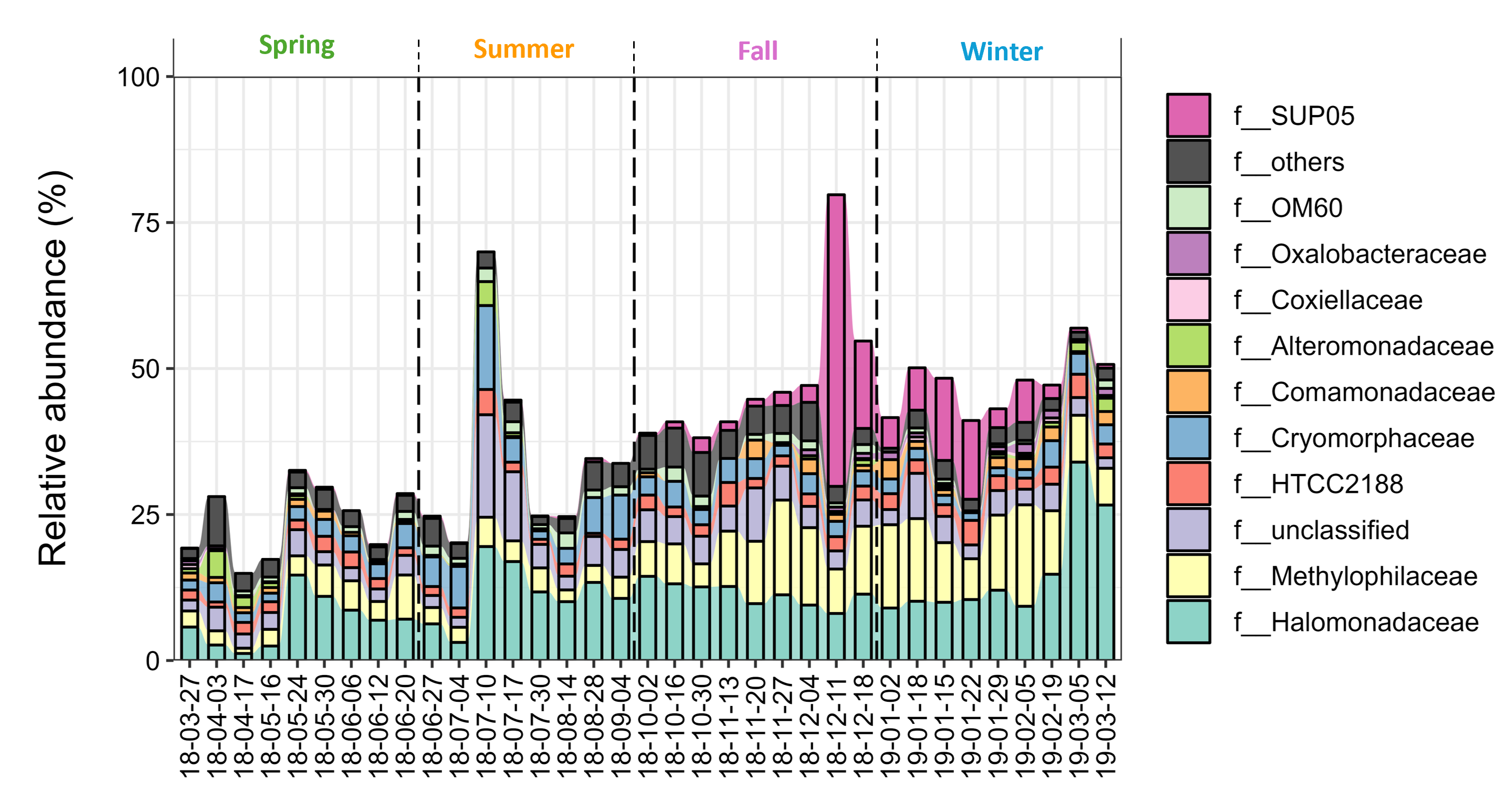


**Figure S4** Relative abundance of taxa categorized as potentially pathogenic phenotype by BugBase. Note: These taxa were assigned against Greengenes database gg_13_5, where *SUP05* was classified at the family level. However, in the SILVA 138.1 database, *SUP05* was classified at the genus level (See details in Table S4).

**Table S6** Water quality monitoring for coastal water at Oostende – Dunes and Seas (51°14′34′′N, 2°56′12′′E; approximately 360 m from our seawater collection site) conducted by the Flemish Environment Agency and the Department of Care from March 2018 to March 2019.

- The data were downloaded from [https://kwaliteitzwemwater.be](https://kwaliteitzwemwater.be)).
- According to the European Bathing Water Directive (2006/7/EC), the classification of ‘Very good’ for coastal water quality indicates that intestinal *enterococci* levels are ≤200 CFU/100 mL, E. coli levels are ≤500 CFU/100 mL, and the swimming advice is ‘No problem’. Reference: <https://kwaliteitzwemwater.be/nl/normen>.

| Date | Water quality | Intestinal *enterococci* (CFU/100 mL) | *E. coli*  (CFU/100 mL) |
| --- | --- | --- | --- |
| 20180522 | Very good | 3 | 27 |
| 20180604 | Very good | 4 | 73 |
| 20180608 | Very good | 19 | 90 |
| 20180612 | Very good | 14 | 34 |
| 20180618 | Very good | 7 | 18 |
| 20180622 | Very good | 10 | 24 |
| 20180626 | Very good | 15 | 25 |
| 20180702 | Very good | 5 | 13 |
| 20180706 | Very good | 2 | 5 |
| 20180710 | Very good | 11 | 20 |
| 20180716 | Very good | 3 | 6 |
| 20180720 | Very good | 1 | 19 |
| 20180724 | Very good | 15 | 123 |
| 20180730 | Very good | 13 | 25 |
| 20180803 | Very good | 2 | 14 |
| 20180807 | Very good | 11 | 10 |
| 20180813 | Very good | 108 | 216 |
| 20180817 | Very good | 19 | 56 |
| 20180821 | Very good | 8 | 21 |
| 20180827 | Very good | 2 | 8 |
| 20180831 | Very good | 17 | 206 |
| 20180910 | Very good | 15 | 25 |

1. Present address: Witteveen+Bos Belgium N.V., Posthoflei 5-1, 2600 Antwerpen-Berchem, Belgium [↑](#footnote-ref-2)
